# A preliminary study of the mechanism of nitrate-stimulated remarkable increase of rifamycin production in *Amycolatopsis mediterranei* U32 by RNA-seq

**DOI:** 10.1186/s12934-015-0264-y

**Published:** 2015-06-04

**Authors:** Zhi Hui Shao, Shuang Xi Ren, Xin Qiang Liu, Jian Xu, Han Yan, Guo Ping Zhao, Jin Wang

**Affiliations:** CAS Key Laboratory of Synthetic Biology, Institute of Plant Physiology and Ecology, Shanghai Institutes for Biological Sciences, Chinese Academy of Sciences, Shanghai, 20032 China; State Key Laboratory of Bioreactor Engineering, New World Institute of Biotechnology, East China University of Science and Technology, Shanghai, 200237 China; State Key Lab of Genetic Engineering and Center for Synthetic Biology, Department of Microbiology and Microbial Engineering, School of Life Sciences, Fudan University, Shanghai, 200032 China; Shanghai-MOST Key Laboratory of Disease and Health Genomics, Chinese National Human Genome Center at Shanghai, Shanghai, 201203 China; Department of Microbiology and Li KaShing Institute of Health Sciences, The Chinese University of Hong Kong, Prince of Wales Hospital, Shatin, New Territories Hong Kong Sar, China

**Keywords:** *Amycolatopsis mediterranei*, Rifamycin SV, Nitrate, RNA-seq, Transcriptome

## Abstract

**Background:**

Rifamycin is an important antibiotic for the treatment of infectious disease caused by *Mycobacteria tuberculosis*. It was found that in *Amycolatopsis mediterranei* U32, an industrial producer for rifamycin SV, supplementation of nitrate into the medium remarkably stimulated the yield of rifamycin SV. However, the molecular mechanism of this nitrate-mediated stimulation remains unknown.

**Results:**

In this study, RNA-sequencing (RNA-seq) technology was employed for investigation of the genome-wide differential gene expression in U32 cultured with or without nitrate supplementation. In the presence of nitrate, U32 maintained a high transcriptional level of genes both located in the rifamycin biosynthetic cluster and involved in the biosynthesis of rifamycin precursors, including 3-amino-5-dihydroxybenzoic acid, malonyl-CoA and (*S*)-methylmalonyl-CoA. However, when nitrate was omitted from the medium, the transcription of these genes declined sharply during the transition from the mid-logarithmic phase to the early stationary phase. With these understandings, one may easily propose that nitrate stimulates the rifamycin SV production through increasing both the precursors supply and the enzymes for rifamycin biosynthesis.

**Conclusion:**

It is the first time to thoroughly illustrate the mechanism of the nitrate-mediated stimulation of rifamycin production at the transcriptional level, which may facilitate improvement of the industrial production of rifamycin SV, e.g. through optimizing the global rifamycin biosynthetic pathways on the basis of RNA-seq data.

**Electronic supplementary material:**

The online version of this article (doi:10.1186/s12934-015-0264-y) contains supplementary material, which is available to authorized users.

## Background

*Amycolatopsis mediterranei* U32 is an important industrial strain for production of rifamycin SV, whose derivative, rifampicin is one of the most commonly used drugs for curing mycobacterial infections [1]. It was previously found that addition of nitrate into the fermentation medium remarkably stimulated the production of rifamycin SV by 170% (abbreviated as the “nitrate stimulating effect” [2]), along with many other global physiological effects, including improving the enzymatic activities of nitrate/nitrite reductases (NR/NiR), glutamine synthetase (GS) and a few intermediate metabolic enzymes and decreasing the intracellular amount of fatty acids [2]. Besides of rifamycin SV, this “nitrate stimulating effect” is also widely applied in industrial production of many other antibiotics, e.g. lincomycin and lividomycin [3, 4]. And because of its great importance in industrial application, research into the molecular mechanism of “nitrate stimulating effect” has never stopped [2, 4–12].

Earlier interests and intensive investigation mainly focused on identification of genes involved in the effect, many of which were related to nitrogen metabolisms [8–10, 13, 14]. With the supplementation of nitrate, U32 mainly uses the GS/GOGAT (glutamate synthase, GOGAT) pathway for nitrogen assimilation. While under nitrogen excess, i.e. with large amount of ammonia, the alanine dehydrogenase (AlaDH, encoded by *ald*) instead of the commonly used glutamate dehydrogenase is adopted for ammonium assimilation [11]. It is found that nitrate increases the GS activities but decreases the activities of AlaDH. Meanwhile, the yield of rifamycin SV is positively correlated with the activities of GS but negatively correlated with the activities of AlaDH. Unlike many other actinomycetes that possess more than one GS encoding gene [15], *glnA* is the only gene that encodes biologically functional GS in U32 [12]. Consistent with the measurements of enzymatic activities, nitrate remarkably activates *glnA* transcription while represses *ald* transcription, and this transcriptional regulation is conducted by GlnR, which is a central regulator for nitrogen metabolism in U32 [8, 10, 11, 16].

In addition, GS in U32 is regulated not at the posttranslational level, i.e. by adenylylation [12], but at the transcriptional level in response to nitrogen availability [5, 16]. Besides *glnA*, nitrate also regulates the transcription many other nitrogen metabolism associated genes in U32 [8, 10, 11, 16]. However, apart from nitrogen metabolism, little is known about the nitrate-mediated global transcriptional network and the detailed mechanism of nitrate mediated stimulation of rifamycin SV production.

Transcriptome analysis, which studies all RNA transcripts under a particular physiological condition, has played an important role in detecting the global gene expression and transcriptional regulation [17]. RNA-sequencing (RNA-seq) technology uses deep-sequencing technologies to directly determine the cDNA sequence and owns single-base resolution, allowing for the detection of various transcriptional features of transcript structure, operon linkages and absolute abundance [18, 19]. In this work, RNA-seq was used to study the transcriptomic change of *A. mediterranei* U32 in response to nitrate supplementation. Total RNAs from two representative time points were analyzed, including the 24 h representing the mid-logarithmic (ML) phase and the 48 h representing the early stationary (ES) phase. Real-time Reverse Transcription-Polymerase Chain Reaction (RT-PCR) was further employed to quantitatively measure the expression of several important functional genes, e.g. genes responsible for both the rifamycin SV precursors supply and its biosynthesis. Based on these findings, a mechanism was finally proposed for the “nitrate stimulating effect” subject to further investigation.

## Results and discussion

### Influence of nitrate supplementation on both physiology and rifamycin SV production of *A. mediterranei* U32

**Figure 1:**
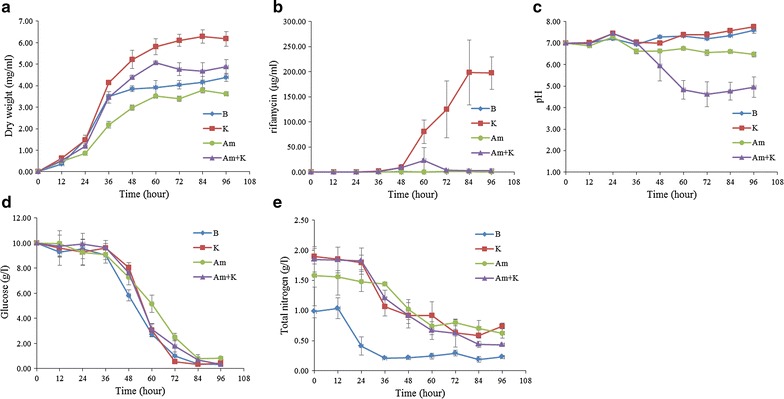
The growth curve (**a**), rifamycin SV production curve (**b**), pH value (**c**), the concentration of glucose (**d**) and total nitrogen (**e**) of *A. mediterranei* U32 in Bennet medium or Bennet with different extracellular nitrogen sources. Symbols used: *B* Bennet medium; *K* Bennet medium with 80 mM KNO_3_; *Am* Bennet medium with 30 mM (NH_4_)_2_SO_4_; *Am* *+* *K* Bennet medium with 20 mM (NH_4_)_2_SO_4_ and 40 mM KNO_3_. Three independent samples were measured.

To study the influence of extracellular nitrogen sources on rifamycin SV production, *A. mediterranei* U32 were cultured in nutrient-rich liquid Bennet medium with or without extracellular nitrogen sources supplemented (Figure 1). Three different nitrogen sources were tested, including 80 mM nitrate, 60 mM ammonium and 80 mM mixed ammonium and nitrate. According to the growth curves, in comparison with the growth in Bennet medium, addition of ammonium slightly repressed the bacterial growth, while both nitrate and the mixed nitrogen source promoted the bacterial growth. However, only nitrate was found to remarkably stimulate the yield of rifamycin SV, which was consistent with our previous findings [2]. Obviously, the total nitrogen could not be the limiting factor for the rate of bacterial growth and rifamycin SV production, because in the conditions of ammonium and mixed nitrogen supplementation, the total nitrogen as well as its consumption rate were the same as those under the condition of nitrate addition (Figure 1e).

In addition, the glucose consumption rate and pH value in the medium were also measured. For the glucose consumption rate, no significant difference could be found among different culture media. The pH values were almost unchanged in all tested conditions except the mixed nitrogen source, in which the pH value approximately decreased from 7 to 5 (Figure 1c). It is still unclear whether the decrease of pH value has any influence on the biosynthesis of rifamycin SV.

In the presence of nitrate, the growth of U32 could be divided into two distinct developmental stages, i.e. the primary metabolism stage for vegetative growth (from 12 to 36 h) and the secondary metabolism stage for production of rifamycin SV (from 36 to 72 h) (Figure 1). To quantitatively measure the contribution of biomass increase and global metabolic regulation in the increase of the rifamycin SV yield, the values of specific production rate (*q*_*P*_) of rifamycin SV between Bennet and Bennet supplemented with nitrate were calculated. When fermentation process finished, the overall *q*_*P*_ value (mg_product_/(mg_dry weight_·h)) was 0.50 for U32 grown with nitrate, while was only 0.003 when nitrate was omitted from the medium. Therefore, nitrate stimulates the production of rifamycin SV mainly through regulation of its production rate other than by increasing the bacterial biomass.

### RNA-seq analysis of the transcriptome of U32 grown in Bennet medium with or without nitrate supplementation

RNA-seq was employed to reveal the nitrate-induced transcriptomic changes in U32. Samples were taken at 24 and 48 h, representing the ML and ES phases, respectively. Before RNA-seq, ribosomal RNA was successfully removed with high mapping ratio obtained (Table 1). Annotated genes include 9228 CDSs, 12 rDNA genes and 52 tRNA-encoding genes [9] and nearly 70% of them expressing with RPKM >5 at two tested time points (i.e. ML and ES phases) either with or without nitrate supplementation (Table 1). On the basis of RNA-seq data, re-annotation of U32 genome information was performed, with the newly identification of the Valyl-tRNA synthetase encoding gene (*AMED_6793.1*) that was missed from previous annotations [9]. In addition, with the use of Rockhopper [20, 21] by setting reads more than 10 and length between 50 and 500 nucleotides, we identified a large number of noncoding RNAs (ncRNAs), including 600 intergenic transcripts and 582 antisense transcripts, which comprised of about 11.29% of total transcripts. It is well-known that ncRNAs are involved in the regulation of various processes such as transcriptional interference, translational control and regulation of mRNA half-life [22, 23]. In U32, the specific physiological functions of these ncRNAs, especially in regulation of secondary metabolisms, are unclear, and it is an interesting direction to delineate their physiological roles in future studies.

**Table 1:** Transcriptomic profiles of *A. mediterranei* U32 in different culture conditions

Sample^a^	Raw reads	Mapped reads	Mapping ratio (%)	Genes (RPKM >5)	Expression ratio (%)	Differential genes^b^
24B	18,335,500	17,101,379	93.27	6479	69.72	219 up/334 down
24K	17,343,634	16,514,363	95.22	6498	69.92	
48B	18,740,965	17,564,533	93.72	6324	68.05	1,033 up/847 down
48K	27,441,452	24,162,480	88.05	6483	69.76	

^a^24B, 24 h in Bennet medium; 24K, 24 h in Bennet medium with 80 mM nitrate; 48B, 48 h in Bennet medium; 48K, 48 h in Bennet medium with 80 mM nitrate. Mapping ratio: mapped reads/raw reads. Expression ratio: genes (RPKM ≥5)/9,293 genes.

^b^Genes expressed under nitrate conditions were compared to those in Bennet medium.

**Figure 2:**
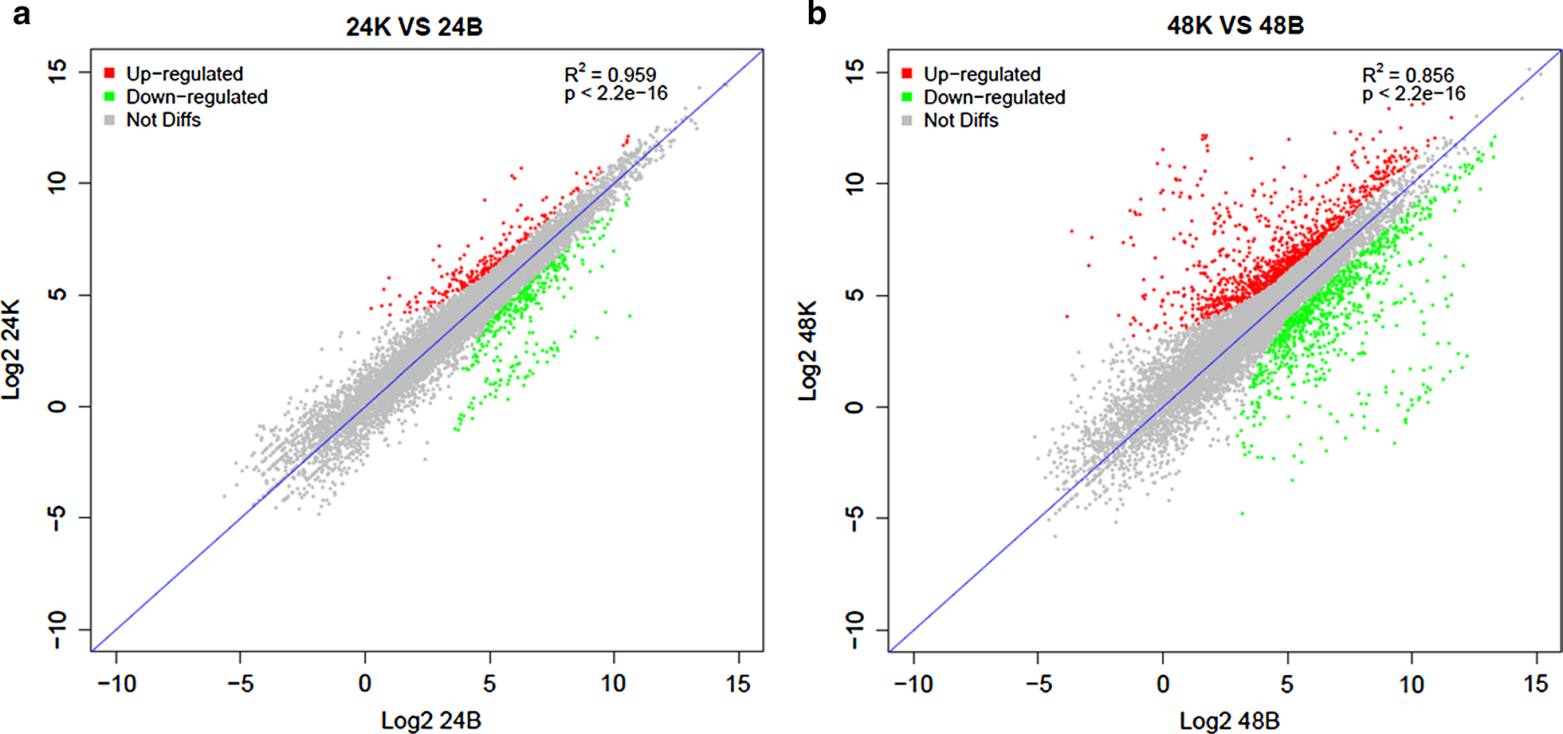
Correlation of RNA expression levels between two nitrogen conditions in two phases of U32. RNA expression levels per gene were presented as RPKM. **a** Correlation of RNA expression levels in 24 h (ML phase) for U32 in Bennet medium with (*K*) or without KNO_3_ (*B*); **b** correlation of RNA expression levels in 48 h (ES phase). Test was considered to be significant when *p* value was <0.05.

In response to nitrate supplementation, both the number and fold change of differentially expressed genes in ES phase were apparently higher than those in ML phase (Figure 2). For example, using statistical criteria of FDR <0.05 (FDR, false discovery rate control) from Fisher test and FC >2 (FC, fold change), there were totally 1,880 genes transcriptionally regulated in ES phase, while only 553 genes were found in ML phase (Table 1). Actually, actinomycetes such as *A. mediterranei* usually transit from the primary metabolism into the secondary metabolism in ES phase and this transcriptionally regulatory pattern at the two growth phases was likely to be consistent with the physiological changes.

According to clusters of orthologous group (COG), functional genes in U32 were divided into 24 categories [9]. Using the Wilcoxon test, 8 categories obviously changed at the transcriptional level in ML phase and 13 categories changed in ES phase in response to nitrate supplementation (Table 2). Consistent with the bacterial physiological growth, the transcriptionally changed categories were mainly related to the primary metabolisms in ML phase, while differential expression of genes related to secondary metabolisms such as the biosynthesis of rifamycin SV were observed in ES phase. Therefore, comparative analyses of the global transcriptional profiling in ES phase, i.e. with versus without nitrate supplementation, were performed in this work to reveal the molecular mechanisms of the nitrate-mediated stimulation of rifamycin SV production.

**Table 2:** Wilcoxon test-assisted statistical analysis of the expression of grouped genes according COG

COG	Description	The number of genes	P value	
			24 h	48 h
A	RNA processing and modification	2	0.5000	0.5000
B	Chromatin structure and dynamics	2	1.0000	1.0000
C	Energy production and conversion	711	0.7816	0.2295
D	Cell cycle control, cell division, chromosome partitioning	55	0.9407	0.0057*
E	Amino acid transport and metabolism	1,072	0.0003*	<0.0001*
F	Nucleotide transport and metabolism	232	<0.0001*	<0.0001*
G	Carbohydrate transport and metabolism	889	0.0012*	0.6980
H	Coenzyme transport and metabolism	475	0.9892	0.1238
I	Lipid transport and metabolism	637	0.9888	<0.0001*
J	Translation, ribosomal structure and biogenesis	268	<0.0001*	<0.0001*
K	Transcription	1,155	<0.0001*	<0.0001*
L	Replication, recombination and repair	265	<0.0001*	0.0085*
M	Cell wall\membrane\envelope biogenesis	515	0.1580	0.0056*
N	Cell motility	43	0.0589	0.2363
O	Posttranslational modification, protein turnover, chaperones	285	0.5207	0.1124
P	Inorganic ion transport and metabolism	544	0.0040*	<0.0001*
Q	Secondary metabolites biosynthesis, transport and catabolism	636	0.2382	<0.0001*
R	General function prediction only	1,646	0.0028*	0.0017*
S	Function unknown	571	0.6361	0.0007*
T	Signal transduction mechanisms	540	0.3348	0.0014*
U	Intracellular trafficking, secretion, and vesicular transport	58	0.7051	0.5160
V	Defense mechanisms	201	0.0637	0.6362
W	Extracellular structures	1	1.0000	1.0000
Z	Cytoskeleton	3	0.5000	1.0000

* Difference is regarded as significant when P value is smaller than 0.01.

### Nitrate supplementation stimulates the precursor supplies in rifamycin SV biosynthesis

**Figure 3:**
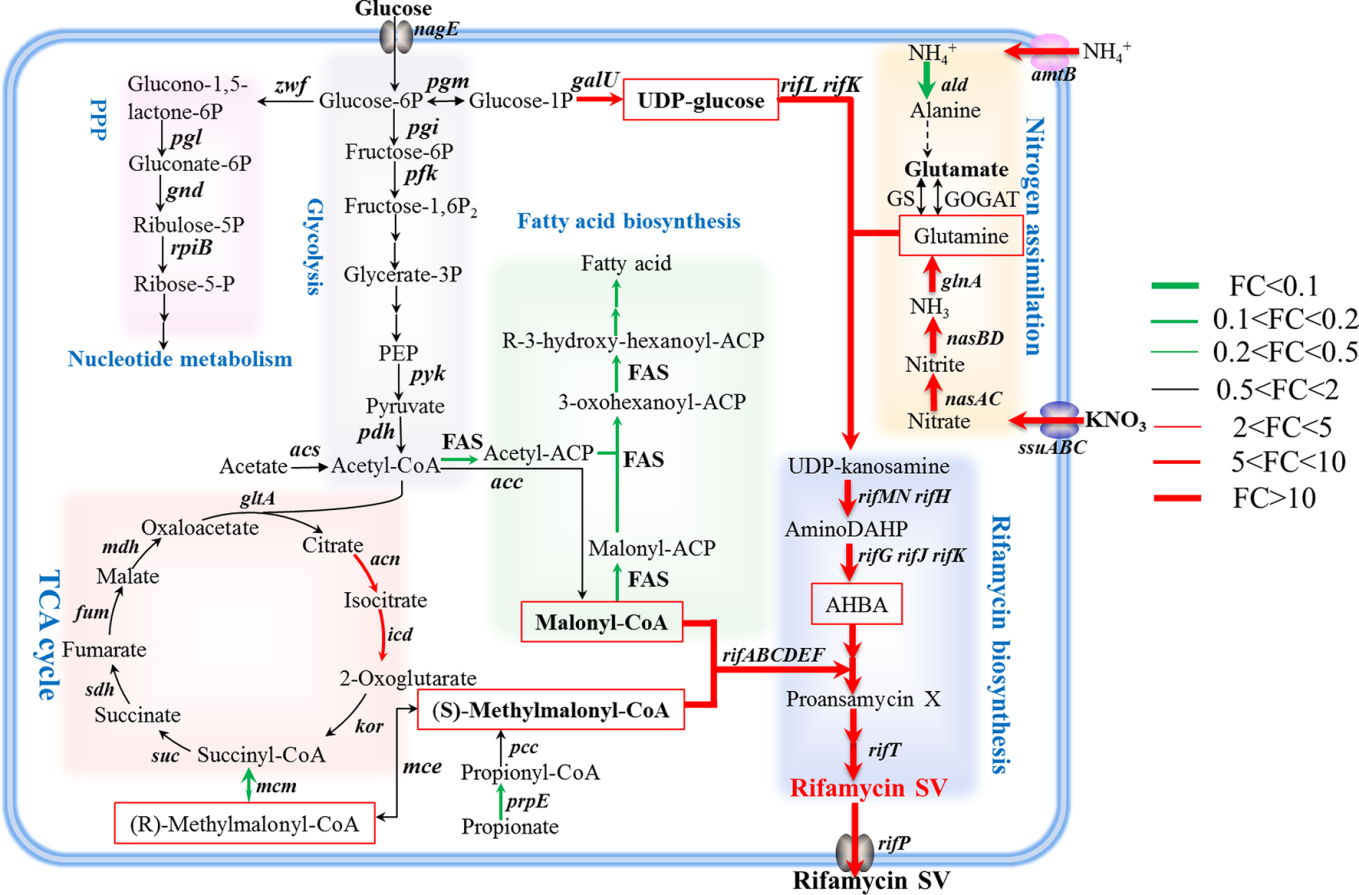
Comparison of the expression of genes involved in carbon and nitrogen metabolisms and rifamycin SV biosynthesis pathways of U32 cultivated in Bennet medium with or without 80 mM KNO_3_ in ES phase. *Solid arrow* one-step reaction; *Double arrow* more than one-step reactions; *dashed arrow* reactions need to be further confirmed. Precursors directly related to rifamycin SV biosynthesis are shown in *red boxes*. *Red arrows* represent up-regulation (FC value >2); *green arrows* represent down-regulation (FC value <0.5); *black arrows* represent the fold changes between 0.5 and 2.

Genes responsible for the biosynthesis of key precursors for rifamycin SV, including 3-amino-5-dihydroxybenzoic acid (AHBA), malonyl-CoA and (*S*)-methylmalonyl-CoA, were significantly regulated in ES phase responding to nitrate supplementation (Figure 3).

AHBA is synthesized step-by-step via enzymes encoded by genes of *rifGHIJKLMN* and *AMED_0651*-*AMED_0652*, employing substrates of UDP-glucose and glutamine. Upon nitrate addition, the transcription of these genes was up-regulated to 24.6, 25.5, 25.5, 55.9, 466.3, 237.0, 24.5, 10.4, 1,354.0 and 977.7 folds, respectively (Additional file 1: Table S1). Compared to conditions without nitrate supplementation, nitrate increased the expression of *galU* gene, which encodes the UDP-glucose pyrophosphorylase catalyzing the biosynthesis of UDP-glucose, by 6.8 folds (Figure 3). Meanwhile, the synthesis of glutamine, which is a key substrate for AHBA biosynthesis and provides the only nitrogen atom in rifamycin SV, was also remarkably enhanced by addition of nitrate. Genes involved in this process were activated at the transcriptional level, including the *nasACKBDE* operon encoding proteins responsible for nitrate assimilation and *glnA* encoding the only GS with biological functions [8, 9, 24] (Additional file 1: Table S1; Figure 3). Nitrate activated the expression of *nasACKBDE* operon and *glnA*, i.e. by 1,399.4, 2,910.6, 779.0, 1,189.1, 465.5 and 463.3 folds for *nasACKBDE* genes and by 13.0 folds for *glnA* (Figure 3), which was in well consistence with previous findings [8, 16].

In addition, totally two malonyl-CoA molecules (C2 extender unit) and eight (*S*)-methylmalonyl-CoA (C3 extender unit) are needed for polyketide chain elongation during the biosynthesis of rifamycin SV [2]. In U32, malonyl-CoA is reversibly converted from acetyl-CoA by the acetyl-CoA carboxylase (Acc). Upon supplementation of nitrate, no obvious difference was found in the expression of *acc* genes. However, the expression of the fatty acid synthase (FAS), which is a hexamer complex encoded by the *fab* operon, was down regulated after nitrate supplementation. In particular, the FabG-encoding genes, *AMED_5426* and *AMED_5430*, both of which expressed more than tenfold higher than other *fab* genes in ES phase (Additional file 1: Table S1), were transcriptionally repressed by threefold after addition of nitrate. As the fatty acids and rifamycin SV share the same common precursor of malonyl-CoA, one may easily propose that nitrate addition shifts the malonyl-CoA flux from the fatty acid biosynthesis to rifamycin SV biosynthesis. Meanwhile, these data were consistent with our previous findings that nitrate repressed the intracellular lipid biosynthesis in *A. mediterranei* when observed with electron microscopy [2].

There are totally three pathways for the biosynthesis of C3 extender unit of (*S*)-methylmalonyl-CoA, including the propionyl-CoA carboxylase (Pcc), methylmalonyl-CoA carboxyltransferase (Mct) and methylmalonyl-CoA mutase (Mcm)–methylmalonyl-CoA epimerase (Mce) pathways. Based on previous enzymatic studies [25], the Mcm–Mce pathway is the main pathway for C3 extender unit biosynthesis in U32. Consistently, no transcriptional difference was observed for Pcc and Mct encoding genes, both of which were annotated as *pcc* or *acc* genes, under two culture conditions.

For the Mcm–Mce pathway, succinyl-CoA is firstly reversibly catalysed into (*R*)-methylmalonyl-CoA by Mcm [26], which is then converted into (*S*)-methylmalonyl-CoA by Mce. There are totally four *mcm* genes annotated in U32, including *AMED_0913*, *AMED_0914*, *AMED_2498* and *AMED_7761*, among which *AMED_2498* was apparently transcriptionally repressed by as much as 25 folds after the addition of nitrate while the transcription of others was almost unchanged in ES phase (Additional file 1: Table S1). The *mutB2*, encoding the large subunit of Mcm, is the key gene for accumulation of intracellular methylmalonyl-CoA [27, 28] and mutation of *mutB2* in *A. mediterranei* HP-130, an overproducer of rifamycin B, significantly increased the yield [29]. Notably, the *mutB2* homologue, *AMED_7761* kept an extremely low transcriptional level in U32, which may account for the high yield of rifamycin SV. Moreover, the transcription of *mce* was up-regulated 1.4 folds upon addition of nitrate, which would therefore lead to enhanced synthesis of the C3 extender unit, (*S*)-methylmalonyl-CoA.

### Nitrate supplementation activates the transcription of the rifamycin SV biosynthetic genes

**Figure 4:**
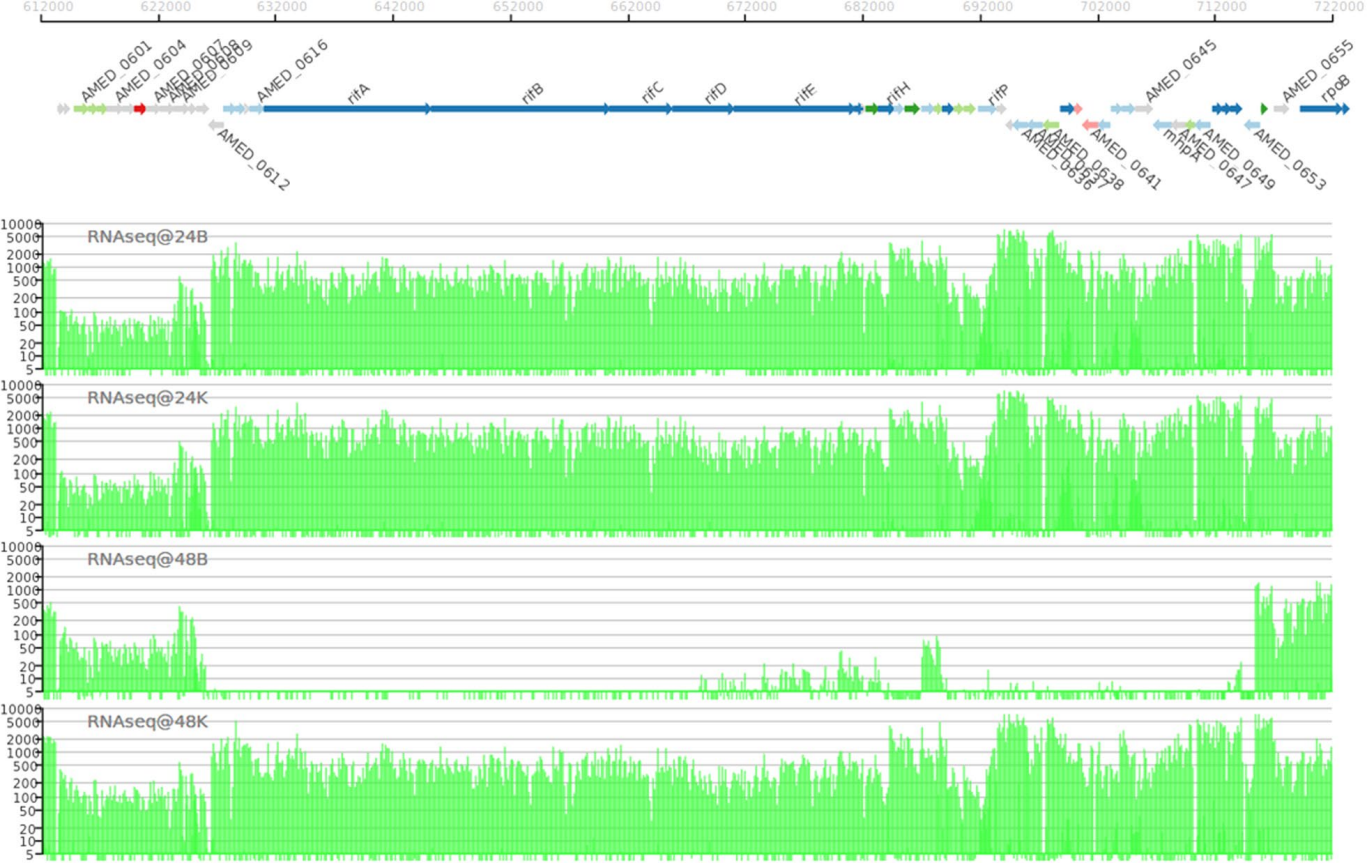
RNA-seq data of rifamycin SV biosynthesis gene cluster, ranging from *AMED_0601* to *AMED_0656* of U32 in Bennet medium with (*K*) or without KNO_3_ (*B*). 24, ML phase; 48, ES phase.

**Figure 5:**
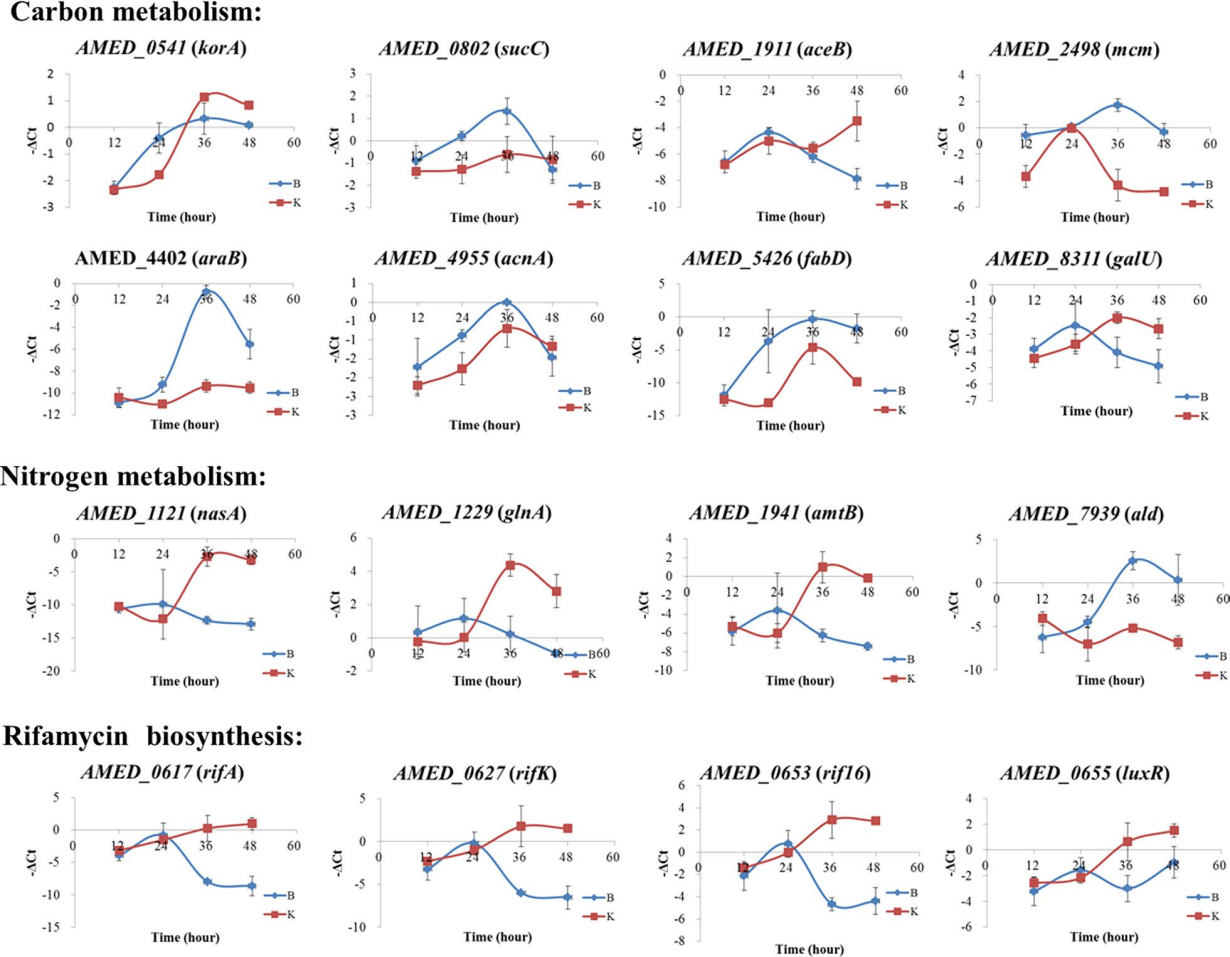
Time-course results of real-time PCR of key genes involved in rifamycin SV biosynthesis for U32 cultivated in Bennet medium with (*K*) or without KNO_3_ (*B*). The transcription of *rpoB* is used as the internal control and error bars represent the standard deviation of three biological replicates.

Besides of the AHBA biosynthetic genes, i.e*. rifG* through *rifN* [30, 31], the transcripts of other important genes in *rif* cluster in ES phase raised several folds (ranging from 7.6 to 2,960.3 folds) higher in nitrate conditions than those in the absence of nitrate. As the products of these genes are responsible for the biosynthesis of rifamycin SV, e.g. *rifABCDE* encode a type I polyketide synthase (PKS) module; several genes encode post-PKS modification enzymes and *rifP* encodes the rifamycin exporter [32, 33] (Figures 4, 5), nitrate supplementation apparently increases the rifamycin SV biosynthetic enzymes.

To validate the RNA-seq data, real-time RT-PCR was used to quantitatively measure the expression of genes involved in both precursors supply and rifamycin SV biosynthetic enzymes. Selected genes include six genes in carbon metabolism such as *korA*, *sucC*, *mcm*, *acnA*, *fabG* and *galU*, four genes involved in nitrogen metabolism such as *nasA*, *glnA*, *amtB* and *ald*, and four genes involved in rifamycin SV biosynthesis such as *rifA*, *rifK*, *rif16* and *luxR*. Four time points of 12, 24, 36 and 48 h were selected for the real-time RT-PCR analysis with transcriptional results in good accordance with the RNA-seq data (Figure 5).

Therefore, a molecular mechanism has been proposed for the “nitrate stimulating effect”. Nitrate obviously activates the transcription of genes responsible for both the precursors supply and the rifamycin SV biosynthesis, which consequently enhances the yield of rifamycin SV. Similarly, in *A. mediterranei* HP-130, an industrial strain for high yield of rifamycin B, *rif* cluster kept continuously higher expression than its reference strain S699 [29].

Besides the *rif* cluster, the transcription of 19 other gene clusters [9] for secondary metabolisms was also investigated (Additional file 2: Table S2). Among them, 11 clusters were found to transcribe at an extremely low level (RPKM <5). Three clusters were transcriptionally repressed by nitrate addition, including nrps_pks1, nrps8 and nrps9, while only nrps6 and nrps10 were transcriptionally activated. Compared to other actinomycetes, e.g., *Streptomyces coelicolor* [34], there are extremely few highly transcribed clusters in U32 under nitrate supplemented condition, which may therefore favor the production of rifamycin SV. In addition, because genes involved in the Glycolysis pathway and TCA cycle, including *pyc* (3.5 folds), *acn* (2.6 folds), *icd* (2.9 folds) and *aceB* (2.6 folds), were transcriptionally activated by addition of nitrate, the energy supply could also be enhanced under this condition, probably facilitating the biosynthesis of rifamycin SV.

### Future perspectives in studying the mechanism of nitrate-mediated global transcriptional regulation

Nitrate has been considered as a poor nitrogen source for bacteria such as *Escherichia coli*, while for plants and algae [35], it is well known as a preferred nitrogen source [36, 37]. For *A. mediterranei* U32, nitrate activates the transcription of a series of genes involved in nitrogen assimilation, e.g. *glnA*, *nas* operon and *amtB*, while represses the expression of *ald*, even in rich Bennet medium. Therefore, considering the transcriptional regulation of these nitrogen metabolism-related genes, one may conclude that Bennet medium with nitrate supplementation mimics a poor nitrogen source for U32 [11]. However, compared to ammonium, nitrate obviously stimulates the growth of U32 in both rich Bennet medium and minimal medium [7]. Therefore, “how would the poor-nitrogen nitrate promote the growth of U32” would be an interesting question subject to further investigation.

GlnR has been characterized as a central regulator for nitrogen metabolisms in several actinomycetes, with *A. mediterranei* U32 included [10, 11, 16, 38, 39]. The transcriptional regulation of nitrogen metabolisms-related genes, including *glnA*, *nas* operon and *ald*, which are closely responsible for the accumulation of glutamine, is under stringent regulation by GlnR [10, 11, 16], responding to extracellular nitrogen availabilities. However, little is known about the transcriptional regulation of other genes that provide precursors for rifamycin SV biosynthesis, e.g*. fabG*, *mcm* and *galU*. As these genes are involved in important pathways of carbon metabolism but are transcriptionally regulated by extracellular nitrogen sources, research into their transcriptional regulation may provide an important clue for study of the coordination of carbon and nitrogen metabolisms in U32.

In addition, the regulation of the transcription of *rif* cluster seems more complex. In ML phase, the transcription of *rif* genes remains at a high level with or without the supplementation of extracellular nitrate. However, when cells enter the ES phase, the transcription of *rif* genes only slightly increased under the condition of nitrate, but decreased dramatically in the absence of nitrate (Figures 4, 5). Therefore, nitrate either prolongs the transcriptional period of *rif* cluster till ES phase or merely helps maintain the stability of the *rif* transcripts. And further delineation of the detailed mechanisms, including identification of regulators/proteins involved, would be an interesting project.

Moreover, nitrate-mediated global regulation must be multi-level, which may include transcriptional, translational and post-translational levels. And for those important genes that are not regulated at the transcriptional level, there may be post-transcriptional regulations, which will be a focus of research in our future work.

## Conclusions

In this work, we in-depth studied the “nitrate-stimulating effect” in U32 at the transcriptional level, based on which a molecular mechanism was successfully proposed. We noticed that most genes involving in both the precursor production and rifamycin SV biosynthesis were transcriptionally activated by nitrate addition, well explaining the nitrate-mediated activation of rifamycin SV production.

## Methods

### Strains and culture conditions

*Amycolatopsis mediterranei* U32 (CGMCC 4.5720) was grown aerobically at 30°C in rich Bennet medium (glucose 1.0%, tryptone 0.2%, yeast extract 0.1%, beef extract 0.1%, glycerol 1.0%, w/v, pH 7.0) [2, 40]. After 48 h’s culturing, 5% of U32 cells was inoculated into fresh liquid Bennet medium or Bennet with extracellular nitrogen sources supplemented, i.e. 80 mM potassium nitrate, 30 mM ammonium sulphate [5] or 80 mM mixed nitrate and ammonium. For mixed nitrogen source, 40 mM potassium nitrate and 20 mM ammonium sulphate were used to make up 80 mM nitrogen. Cells were harvested at different time points (12, 24, 36 and 48 h) by centrifugation at 12,000 rpm for 5 min at 4°C and were then quickly frozen in liquid nitrogen for total RNA isolation.

### Measurement of growth rate, rifamycin SV production, pH value, glucose consumption and total nitrogen in medium

The growth curves were determined through measuring the bacterial dry weight. The pH value in the medium was measured by a pH meter (Mettler Toledo). The glucose concentration was determined with an enzymatic test kit (Rsbio, Shanghai, China), following the manufacturer’s instructions. The total nitrogen concentration (as nitrate-N) was measured with a colorimetric way, the same as that described by Ferree et al. [41].

Rifamycin SV was measured as previously described [9, 13]. Strains were cultured for 4 days in Bennet liquid medium at 30°C. Samples of the culture broths were taken with every 24 h internals, adjusted to pH 2–3 with 1 M HCl and extracted once with equal volumes of ethyl acetate. Ethyl acetate solutions were filtered (0.22 μm) and then directly analyzed by HPLC (Agilent HPLC 1260 Infinity) with detection wavelength at 280 and 447 nm, employing the 95% pure rifamycin SV (Sigma-Aldrich) as a reference standard. The column was an Agilent poroshell 120 EC-C18 column (4.6 × 50 mm, 2.7 mm, 30°C) and the mobile phase was 70% methanol in water supplemented with 0.1% formic acid with a flow rate of 1.0 ml/min. All data were presented as mean ± standard deviations for the number of experiments indicated in each case.

The values of specific production rate (*q*_*P*_: mg_product_/(mg_dry weight_·h))of rifamycin SV between two growth conditions were calculated according to the following Eqs. (1) and (2) [*ρ*_*X*_: cell concentration (mg/ml); *ρ*_*P*_: product concentration (mg/ml); t: culture time (h)]

1 $$ q_{P} = \frac{1}{{\rho_{X} }} \cdot \frac{{d\rho_{P} }}{dt} $$

2 $$ q_{P} = \frac{{\rho_{P j} }}{{\mathop \sum \nolimits_{i = 1}^{j} \frac{{\rho_{x i} + \rho_{x (i - 1)} }}{2} \times (t_{i} - t_{i - 1} )}}                      j = \frac{t}{12} $$

### Sample preparation for RNA-seq and sequencing procedures

Bacteria were lysed by constantly grinding in the presence of liquid nitrogen and total RNA was extracted using TRIzol reagent (Invitrogen). RNA was fully treated with RNase-free DNase I (Takara) to prevent contamination of trace genomic DNA [8, 42]. The RNA quality was evaluated by the BioAnalyzer 2100 system (Agilent) and ribosomal RNAs were removed with the RiboZero rRNA removal kit (Epicenter) for gram-positive organisms prior to sequencing analysis. After deprivation of the rRNA, RNA was fragmented and used as a template for a randomly primed PCR.

Strand-specific cDNA libraries were prepared by standard techniques for subsequent Illumina sequencing using the mRNA-seq Sample Prep kit (Illumina). The concentration of the cDNA library was detected by Qubit®2.0 Fluorometer and verified for appropriate fragment size (200–300 bp) on a BioAnalyzer 2100 system (Agilent). Samples were amplified onto flowcells using an Illumina cBot and sequenced on an Illumina HiSeq 2500 for 51 cycles according to manufacturer protocols. Raw sequencing data was processed using the data collection software provided by Illumina [43].

### Data analysis for RNA-seq

Sequencing raw reads were preprocessed by filtering out rRNA reads, sequencing adapters, short-fragment reads and other low-quality reads. The rest reads were used to map to reference genome of *A. mediterranei* U32 at the NCBI website by Bowtie2 software (version: 2.0.5), based on the local alignment algorithm [43]. Reads aligning to multiple locations are kept (to a maximum of 20 potential positions) to assist constructing gene models for genes with repetitive or low complexity features. When aligning reads, 1 mismatch to the reference was allowed. Alignments reported from Bowtie2 software were further processed by the BEDTools software [44] to determine the quantity of the expression of transcripts and their differential expression between conditions. Expression values were presented as Reads Per Kilobase of Gene Per Million Mapped Reads (RPKM). Small non-coding RNAs were identified by Rockhopper software [20, 21]. Data were visualized using the Integrated Genomics Viewer. Differential expression of all of transcripts was quantified through DEGseq software (version: 2.16.1) [45], and then the values of fold change were represented. In general, it was regarded as no distinct difference between two transcripts when their value of fold change fell in between 0.5 and 2. At the same time, P-values were determined and significance was accurately assessed by conducting correction for multiple testing such as Fisher test and Wilcoxon test. RNA-seq data have been submitted to NCBI (accession number: GSE63313).

### Quantitative real-time PCR

Reverse transcription was performed with a random hexamer primer using 2 µg total RNA in a volume of 20 µl employing SuperScript III reverse transcriptase (Invitrogen). PCR was performed employing 20 ng reaction mixtures as the template, using the *rpoB* gene as the internal control. A negative control was performed by following the same procedures except that the addition of reverse transcriptase was omitted. Totally, 14 genes, including 6 genes related to carbon metabolisms, 4 genes related to nitrogen metabolism and 4 genes involved in rifamycin biosynthesis according to RNA-seq data, were selected for quantitative real-time PCR (qPCR) validation experiments, using a SYBR Premix Ex Taq GC kit (Takara) in a Step-one Plus real-time PCR system (Applied Biosystems).

## Additional files

Additional file 1: Table S1. Transcriptional profiles of genes involved in carbon metabolism, nitrogen metabolism and rifamycin SV biosynthesis in *A. mediterranei* U32. Additional file 2: Table S2. Transcriptional profiles of genes for secondary metabolisms except for the rifamycin SV biosynthesis in *A. mediterranei* U32.

## Abbreviations

RNA-seq: RNA-sequencing
NR/NiR: nitrate/nitrite reductases
GS: glutamine synthetase
GOGAT: glutamate synthase
AlaDH: alanine dehydrogenase
RT-PCR: Reverse Transcription-Polymerase Chain Reaction
ML: mid-logarithmic
ES: early stationary
AHBA: 3-amino-5-dihydroxybenzoic acid
Acc: acetyl-CoA carboxylase
FAS: fatty acid synthase
Pcc: propionyl-CoA carboxylase
Mct: methylmalonyl-CoA carboxyltransferase
Mcm: methylmalonyl-CoA mutase
Mce: methylmalonyl-CoA epimerase
*Sdh*: succinate dehydrogenase
*Suc*: succinyl-CoA synthetase
PKS: polyketide synthase
PEP: phosphoenolpyruvic acid
aminoDAHP: 3,4-dideoxy-4-amino-d-arabino-heptulosonic acid 7-phosphate
